# Effects of Graphene-Based Nanomaterials on Microorganisms and Soil Microbial Communities

**DOI:** 10.3390/microorganisms12040814

**Published:** 2024-04-17

**Authors:** Huifen Cao, Xiao Zhang, Haiyan Wang, Baopeng Ding, Sai Ge, Jianguo Zhao

**Affiliations:** 1 College of Agriculture and Life Science, Shanxi Datong University, Datong 037009, China; caohuifen277@126.com; 2 Engineering Research Center of Coal-Based Ecological Carbon Sequestration Technology of the Ministry of Education, Key Laboratory of Graphene Forestry Application of National Forest and Grass Administration, Shanxi Datong University, Datong 037009, China; dingbp@sxdtdx.edu.cn (B.D.); jgzhaoshi@126.com (J.Z.); 3 College of Chemistry and Chemical Engineering, Shanxi Datong University, Datong 037009, China; 4 Center of Academic Journal, Shanxi Datong University, Datong 037009, China; gs_sxdtdx@163.com

**Keywords:** graphene-based nanomaterials, antimicrobial, soil microbial community

## Abstract

The past decades have witnessed intensive research on the biological effects of graphene-based nanomaterials (GBNs) and the application of GBNs in different fields. The published literature shows that GBNs exhibit inhibitory effects on almost all microorganisms under pure culture conditions, and that this inhibitory effect is influenced by the microbial species, the GBN’s physicochemical properties, the GBN’s concentration, treatment time, and experimental surroundings. In addition, microorganisms exist in the soil in the form of microbial communities. Considering the complex interactions between different soil components, different microbial communities, and GBNs in the soil environment, the effects of GBNs on soil microbial communities are undoubtedly intertwined. Since bacteria and fungi are major players in terrestrial biogeochemistry, this review focuses on the antibacterial and antifungal performance of GBNs, their antimicrobial mechanisms and influencing factors, as well as the impact of this effect on soil microbial communities. This review will provide a better understanding of the effects of GBNs on microorganisms at both the individual and population scales, thus providing an ecologically safe reference for the release of GBNs to different soil environments.

## 1. Introduction

As the most diverse and abundant organisms on earth, the importance of microorganisms to humans cannot be overemphasized [1]. Unlike plants and animals, most microorganisms are characterized by rapid reproduction and easy spread. At the same time, many microorganisms are pathogens of plants and animals. The alteration of microorganisms and microbial communities often rapidly affects soil, plants, and animals, and even causes great disasters for human beings. Therefore, the effects of any substance on microorganisms must be carefully evaluated before it is released into the environment.

Carbon nanomaterials were regarded as a kind of peculiar engineering nanomaterial composed of carbon atoms as the prime architecture [2]. Different types of carbon nanomaterials have been reported in the literature, such as 0D carbon quantum dots and fullerenes, 1D carbon nanotubes, 2D graphene, and 3D carbon nanoflowers [2,3]. Graphene, which was first obtained by Geim and Novoselov using micromechanical cleavage in 2004 [4], is a wonderful hexagonal honeycomb-structured carbon nanomaterial consisting of one or a few layers of sp^2^-hybridized carbon atoms [5]. Due to the great proportion of the graphene surface being exposed, modifications of different functional groups are often present on its surface or edges to form functionalized graphene [6]. In recent years, graphene-based nanomaterials (GBNs), which are represented by graphene and graphene derivatives (graphene oxide (GO); reduced graphene oxide (rGO); graphene quantum dots (GQD); twisted multilayer graphene; and hydrophobically modified graphene oxide (HGO), etc.) [7,8,9], have been increasingly applied in biomedicine [10], energy [11], agroforestry [12,13], and many other fields. Consequently, large-scale applications and significant environmental releases have raised significant concerns about their biosafety [14].

Currently, a large number of research cases on GBNs’ antibacterial and fungal resistance, and a few studies on the effect of GBN treatment on bacterial and fungal diversity are reported in the literature. Many reviews on GBNs’ antimicrobial effects are available [15,16,17,18,19,20]; these mainly focus on their antimicrobial mechanisms and applications. However, the literature summarizing the knowledge of GBN’s effect on microbial communities is relatively limited [21,22]. This review attempts to summarize the biological effects of GBNs on microorganisms at both the individual and community levels (soil microbial communities), thus providing a more comprehensive analysis of the effects of GBNs on microorganisms.

## 2. The Effects of GBNs on Bacteria and Fungus

As summarized in the literature, the main strategies for determining the antimicrobial activity of exogenous substances include dilution methods, diffusion methods, bioautography methods, time-kill tests, and Adenosine triphosphate (ATP) bioluminescence assays, etc. [23,24]. Methods such as dilution methods and diffusion methods have been extensively used in the study of GBNs’ antimicrobial activity. Bacteria are single-cell structures, which exist in the form of colonies on a solid medium, while the proliferation process in a liquid medium conforms to a growth curve. Therefore, in most studies, after co-culture with GBNs in liquid medium for a certain time, cloning counting and growth curve analysis methods are widely used for quantitative analysis in GBN antibacterial studies. The life cycle of fungi varies from species to species, but there are usually developmental stages such as spore formation, spore germination, and mycelium growth. Thus, parameters measuring the state of spore germination and mycelial growth, such as spore germination rate, mycelial biomass, and colony diameter, were used to evaluate the antifungal performance of GBMs. As with most stimulants, treatment with GBNs will inevitably cause changes in the outer morphology and in the expression of genes and metabolites in microorganisms. Accordingly, the use of scanning electron microscopy technology to observe the surface morphology of microorganisms, and the technology to detect energy metabolism in vivo and analyze gene expression are also widely used in the research process.

In order to analyze the effects of GBNs on microorganisms, in-depth studies have been carried out on several bacterial and fungal species using different types of GBNs. Despite the influence of various factors such as functional modification; microbial species; layer number; and treatment condition, GBNs, including GO, rGO, FLG, HGO, always showed concentration- and time-dependent inhibitory effects on almost all the test bacteria and fungi, which are demonstrated in Table 1 and Table 2, respectively. The bacteria tested were *Escherichia coli* (*E. coli*); *Staphylococcus aureus* (*S. aureus*); *Xanthomonas oryzae pv. Oryzae* (*Xoo*); *Bacillus cereus* (*B. cereus*); *Enterococcus faecalis* (*E. faecalis*); *Pseudomonas syringae* (*P. syringae*); *Xanthomonas campestris pv. undulosa* (*X. campestris pv. undulosa*); and *Pseudomonas aeruginosa* (*P. aeruginosa*). The fungi tested were *Candida albicans* (*C. albicans*); *Fusarium graminearum* (*F. graminearum*); *Bipolaris sorokiniana* (*B. sorokiniana*); *Aspergillus niger* (*A. niger*); *Aspergillus oryzae* (*A. oryzae*); *Fusarium oxysporum* (*F. oxysporum*); *Fusarium poae* (*F. poae*); *Botrytis cinerea* (*B. cinerea*); and *Plasmopara viticola* (*P. viticola*).

## 3. The Mechanism of GBNs’ Antimicrobial Activity

**Figure 1:**
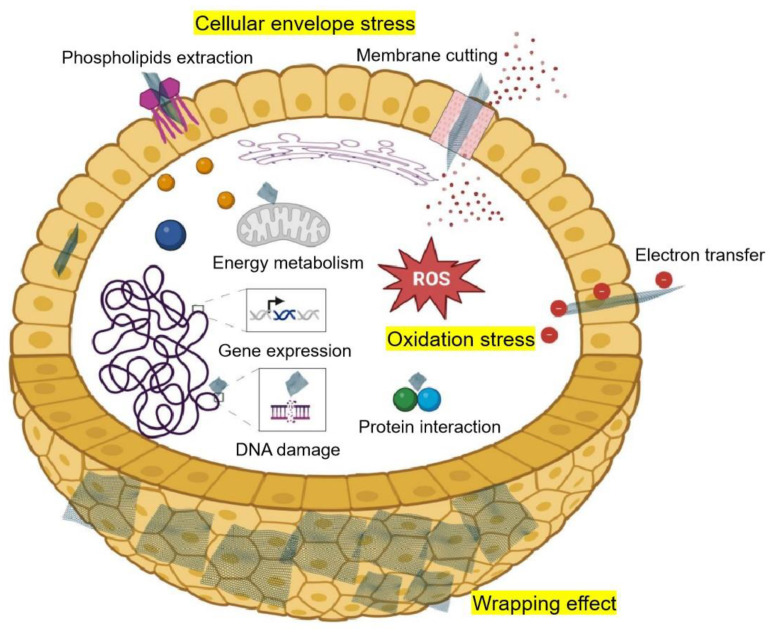
Schematic diagram representing antibacterial mechanisms of GBNs. Created with BioRender.com.

Several mechanisms were proposed to explain GBNs’ antimicrobial activity, which could be summarized in Figure 1 through three effects: cellular envelope stress, oxidation stress, and wrapping effect [16,18,20,42]. At the same time, the interaction between GBNs and microorganisms is closely related to the physical and chemical properties of GBNs, the types of microorganisms, and other factors. At the same time, the antimicrobial activity of GBNs is closely related to the physicochemical properties of GBNs, microbial species, and state [20].

The cellular envelope stress caused by GBNs includes membrane cutting and phospholipid extraction. GBNs generally have thin sheets and very sharp edges, and its direct physical contact with cells easily leads to disruption of the cell membrane system integrity and the leakage of cytoplasmic fluid, thus reducing microorganism viability [28,43]. Because the bond between graphene nanosheets and phospholipid tails is greater than the interaction of the phospholipid molecules, the graphene inserted into the cell membrane extracts a large amount of phospholipid, which induces pore formation on the surface of the cell membrane [44,45]. Actually, the effect of GBNs on the growth of biofilms was not consistent. For example, the biofilm growth of *E. coli* may be either promoted or inhibited by GO treatment [46,47]. In addition, the formation of *E. coli* and *Bacillus subtilis* biofilm could be encouraged by low concentrations of GO, while be inhibited by high concentrations of GO [48].

Oxidative stress, including reactive oxygen species (ROS)-dependent and ROS-independent oxidative stress, is considered to be another important mechanism for the antimicrobial resistance of GBNs [42]. ROS-dependent oxidative stress is caused by the accumulation of large amounts of ROS. Compared to the control cells, the levels of ROS in *P. aeruginosa* cells were elevated 2.8-fold and 1.7-fold after GO and rGO treatment, respectively [31]. An elevated level of ROS in a cell will cause serious damage to phospholipids, DNA, and proteins, and then damage the integrity of the cell membrane system; interfere with gene expression and genetic information transmission; cause the loss of protein function; increase the concentration of intracellular calcium ions; and eventually lead to cell death [15,42]. In addition to ROS-dependent oxidative stress, Li et al. proposed a new electron transfer theory of GBN antibacterial properties, that is, the antibacterial effect of GBNs is mainly due to their super ability to transfer electrons from the microbial membrane [49]. This ROS-independent oxidative stress theory explains the different antibacterial properties of graphene films on conductors and insulators.

Wrapping effect has been proposed as the third important mechanism for GBNs’ antimicrobial activity. The thin and flexible lamellar structure of GBNs enables them to wrap the microbial cell wall, thus becoming an obstacle to the uptake of necessary nutrients from the surrounding environment and eventually inhibiting cell growth or causing cell death [34,50]. 

In addition, GBNs treatment with a high concentration of GO gradually reduced the ATP level and changed the expression of many genes in *R. solanacearum* [33]. In addition, the hydrophobicity of GBNs makes it possible to intervene in the hydrophobic interface of interacting proteins, thereby disrupting protein–protein interactions, which may alter the normal metabolic activities of cells and threaten their survival [51]. Interestingly, GBNs have been shown to enhance the effect of photothermal therapy (PTT)-based cancer treatment due to their intrinsic near-infrared (NIR) absorption properties [52]. Although this remarkable effect was obtained under specific NIR light, the contribution of GBNs to enhancing the antibacterial effect by enhancing NIR absorption under natural conditions needs to be studied in more detail [53].

## 4. The Factors Influencing GBN Antimicrobial Activity

Many factors affect the antimicrobial effect of GBNs. The physicochemical properties of GBNs, which can be described as lateral size, layer number, particle shape, surface modification and dispersion, etc., are dominant factors in determining their antibacterial properties [20,54]. For example, the lateral size of GBNs is proportional to their surface energy, and therefore affects their adsorption [42]. In a certain range, the lateral size of GBNs is positively correlated with their antibacterial activity [25]. GBNs have a lamellar structure composed of a few layers of carbon atoms, and the number of layers obviously greatly affects their thickness, edge morphology, dispersion, and so on. The research from Mangadlao et al. showed that the antibacterial activity of GBNs increased with the increase in lamellar structure [55]. The shape of GBNs largely determines whether they can be easily inserted into cell membranes, and is therefore an important factor in determining their antimicrobial properties. Studies have shown that graphene films with smooth sides have effective bactericidal activity against both *P. aeruginosa* and *S. aureus*, whereas rough sides were only effective in killing *P. aeruginosa* [56]. Primitive graphene tends to agglomerate. In order to increase solubility, different functional groups (e.g., hydroxyl, carboxyl, amino, etc.) are often modified on the edges or inside of graphene [57]. It was experimentally identified that the antibacterial activity of the bacterially reduced GO (BRGO) was distinct from unmodified GO sheets on the activity of *E coli*. [58]. The aggregation conditions of many nanoparticles, such as carbon nanotubes and fullerene, could significantly influence their antibacterial activity [59,60]. According to the report by Liu et al., GO, which has a smaller size and better dispersion than rGO, exhibits stronger antibacterial properties than rGO [27].

The antimicrobial properties of GBNs greatly vary among different microbial species. For instance, Gram-positive bacteria (e.g., *S. aureus* and *E. faecalis*), which have a thicker peptidoglycan layer, were more sensitive to GBNs than Gram-negative bacteria (e.g., *E. coli* and *P. aeruginosa*) [26,28]. Moreover, the physiological state of the microorganism was considered as a key modulator of the GBNs’ antimicrobial activity. Bacteria at different stages of the growth curve were shown to be sensitive to graphene materials in the following order: exponential ≫ decline > stationary [61].

In addition, similar to the effect of GBNs on plant growth [62], the antibacterial properties of GBNs are also concentration-, time- and experimental surroundings-dependent. As with almost all agents, increasing the treatment time and concentration could increase the chances of GBNs interacting with microorganisms. Moreover, many parameters of the experimental surroundings, such as pH values, electricity, magnetic field, light, and sonication conditions, are also important factors in determining the antibacterial activity of GBNs [42]. Interestingly, both GO/rGO dissolved in water and freestanding graphene-based papers have shown antibacterial effects against *E. coli* [63], while 25 μg/mL GO added to Luria Bertani (LB) nutrient broth enhances the cell growth of *E. coli* [64].

## 5. The Effects of GBNs on Soil Microbial Communities

In order to simplify the research, most current research on the interaction between GBNs and microorganisms adopts a single-strain research method under pure culture conditions. However, there are almost no single microbial species in nature, but rather they exist in the form of microbial communities [22]. Compared to the study of the effects of graphene on single microorganisms under pure culture conditions, little is known about the effects of GBNs on microbial communities in soil. We chose the terms “TI = ((graphene and soil and (microbial or fungal or bacterial)))” to cover the literature of this topic in Web of Science™, and identified 22 pertinent publications from 2014 to the time the article was submitted (3 January 2024). We then summarized the relevant literature to describe the effects of GBNs on soil microbial communities in Table 3.

The microbial communities themselves differ greatly in different ecological environments, and the environment also affects the physical and chemical properties of GBNs. In this review, the soil includes urban soil, farmland soil, grassland soil and so on under different climatic and pollution conditions. In addition to the variety of GBNs, exposure times and concentrations in different studies were also varied, giving us the opportunity to understand the short- and long-term effects of different concentrations of GBNs. At present, the research on microbial communities mainly relies on two methods: one is to analyze the total microbial biomass in soil by examining the total organic carbon content in soil, and the other is to analyze the microbial richness and diversity via high-throughput sequencing. 

In contrast to the uniform effect of GBNs on a single microorganism species, their effects on soil microbial communities are very diverse. For instance, the total soil microbial biomass may increase [69], decrease [81], or remain unchanged [65,66] after GBNs treatment. In parallel, GBNs treatment can also affect the microbial richness, with the exception of the lowest concentration on day 14. For example, after 7–30 days of treatment, GBNs treatment changed the microbial community structure, but not the alpha diversity [70,71]. Furthermore, the composition of bacterial and fungal communities was significantly altered by GO at all doses [71]. Specially, some bacterial populations, such as those involved in nitrogen biogeochemical cycles and the degradation of organic compounds, showed significant changes after 4 days of graphene treatment [69]. *Proteobacteria* was the most abundant phylum. *Bacillus*, *Lactococcus*, *Lysobacter, Flavobacterium*, *Pedobacter* and *Massilia* were the most abundant genera under graphene and GO treatments [72]. After exposure to GO and rGO, some of the functional groups associated with organic matter degradation and biogeochemical cycling of nitrogen and sulfur decreased, and the functional group associated with aromatic compound degradation increased [73]. Some nitrogen-fixing and dissimilatory iron-reducing bacteria were selectively enriched after pristine graphene oxide treatment, especially at the genus level [76]. Graphene treatment increased the relative abundance of the majority of bacterial phyla (e.g., *Proteobacteria* and *Actinobacteria*), and decreased the relative abundance of some phyla (e.g., *Verrucomicrobia* and *Acidobacteria*) in Cd-contaminated soil [80].

## 6. Discussion and Conclusions

As the application scenarios of GBNs become more and more abundant, the impact of environmental factors on GBNs themselves and the antimicrobial activity of GBNs will be paid more and more attention. Although the types of GBNs, surface modifications, microbial species, and so on are very diverse, the current generally accepted view is that GBNs exhibit antimicrobial activity under pure culture conditions [15,16].

At high concentrations, GBNs have a fairly high probability of exhibiting toxic effects on different types of organisms and cell lines [82]. As summarized in this paper, the antibacterial effect of GBNs is mainly due to cellular envelope stress, oxidation stress, and wrapping effects caused by GBNs. For animal cells, it is also common for GBNs to induce inflammation, apoptosis, autophagy, and necrosis [83]. On the one hand, the mechanical cutting and phospholipid extraction of GBNs will greatly damage the integrity of a cellular membrane [15]. On the other hand, GBNs entering a cell will pose a great threat to the structure (e.g., DNA integrity) and function (e.g., protein interactions) of macromolecules [42].

Microbial communities exist widely in different environments on earth and play an important role in biogeochemical cycles. The naive idea is that the antimicrobial effects of GBNs will cause them to suppress microbial biomass in the soil, but that is not the entirety of their actual effect. There is both coordination and competition between different populations in microbial communities, and different ecological environments have different degrees of stress effects on them, which makes it very difficult for us to really clarify the effect of GBNs on microbial communities. In particular, in many studies the physical and chemical properties of GBNs were not well tracked and deeply analyzed after their application to soil, which may be the reason for the significant differences in the effects of GBNs in different studies. In addition, in the production and functionalization modification process of GBNs a large number of harmful impurities, such as toxic organic solvents, surfactants, strong acids, and oxidants, will be retained in the final GBNs products to varying degrees, and bring different degrees of toxic effects on microorganisms, which is also one of the reasons for the difference in the biological effects of GBNs in different studies [84,85].

Unlike other compounds (especially organic ones), the stability of GBNs are greatly influenced by environment. Due to the π-π bond between the GBN sheets, Coulombic interactions, and the van der Waals force, it is extremely easy for GBNs to agglomerate [86]. In aqueous environments, it is not only the chemical composition of the GO flakes that determines their morphologies; external factors such as pH and the coexisting cations also influence the structures formed [87]. Soil is a complex colloidal environment. Our previous studies have shown that the biological effects of GO on mung beans vary greatly in aqueous solutions, solid media and soil [62]. This may also imply that the graphene in the pure culture conditions in this paper mainly exhibits antibacterial effects, and the biological effects of graphene in soil are very inconsistent.

Although the industrialization and application of GBNs have developed rapidly, the time and scope of their application are still very limited, so the amount of GBNs in the current environment is very limited. According to Sun et al., the amount of GBNs accumulated in the soil is predicted to increase 5.1 ng/kg each year [88]. However, it is clear that the concentration of GBNs used in the current study is much higher than the real amount in the environment. The current studies on the effects of GBNs on single microbial species or microbial communities are almost all obtained under laboratory conditions. With more environmental releases of GBNs, in situ studies of their effects on microorganisms in more ecological environments will be of great significance.

## Figures and Tables

**Table 1:** Summary of GBNs ability to inhibit bacteria.

Microbial Species	GBNs	Methods	Antimicrobial Performance	Ref.
*E. coli* (G^−^)	GO	Colony counting methods	GO exhibits lateral size-, time- and concentration-dependent antibacterial activity.	[25]
	GO	Viability, time-kill and lactose dehydrogenase release assays	GO exhibits concentration- and time-dependent antibacterial activity.	[26]
	GO, rGO	Colony counting methods	GO and rGO have much higher bacterial inactivation percentages compared with those of Gt and GtO.	[27]
	GO, rGO	Drop-test and colony counting methods	*E. coli* is less sensitive than *S. aureus* to GO and rGO treatment; rGO is more toxic to bacteria than GO.	[28]
	FLG	Bioluminescent signal measure, disk diffusion method	FLG concentration-dependently decreases viable *E. coli*.	[29]
*S. aureus* (G^+^)	GO	Viability, time-kill and lactose dehydrogenase release assays	GO exhibits concentration- and time-dependent antibacterial activity.	[26]
	GO, rGO	Drop-test and colony counting methods	*S. aureus* is more sensitive than *E. coli* to GO and rGO treatment; rGO is more toxic to bacteria than GO.	[28]
	GO, rGO	Growth curve analysis	GO and rGO restrict *S. aureus* cell growth by 93.7% and 67.7%, respectively.	[30]
	FLG	Bioluminescent signal measure, disk diffusion method	Only high concentrations of FLG can lead to decreased viability of *S. aureus*. Both HNO_3_ and H_2_O_2_ in doped FLG-coated textile are capable of inhibiting growth of adjacent bacteria.	[29]
*E. faecalis* (G^+^)	GO	Viability, time-kill and lactose dehydrogenase release assays	GO exhibits concentration- and time-dependent antibacterial activity.	[26]
*P. aeruginosa* (G^−^)	GO	Viability, time-kill and lactose dehydrogenase release assays	GO exhibits concentration- and time-dependent antibacterial activity.	[26]
	GO, rGO	Colony-counting method	GO and rGO show concentration- and time-dependent antibacterial activity against *P. aeruginosa* cells.	[31]
	GO, rGO	Growth curve analysis	GO and rGO restrict *P. aeruginosa* cell growth by 48.6% and 93.3%, respectively.	[30]
	HGO	Diffusion plate method (solid nutrient agar)	Composite film containing 15 wt% HGO is able to develop clear inhibition zone compared to blank polystyrene film.	[32]
*R. solanacearum* (G^−^)	GO	Growth curve analysis	GO suppresses growth of *R. solanacearum* at all test concentrations (ranging from 62.5 to 500 μg/mL) in less than 2 h of incubation time.	[33]
*P. syringae* (G^−^)	GO	Growth curve analysis	GO can significantly inhibit bacterial growth in concentration range 10 to 500 mg/mL.	[34]
*X. campestris pv. undulosa* (G^−^)	GO	Growth curve analysis	GO can significantly inhibit bacterial growth in concentration range 10 to 500 mg/mL.	[34]
*Xoo* (G^−^)	GO, rGO	Colony counting methods	GO and rGO delay growth of *Xoo*, primarily depending on concentration and type of buffer. GO is more toxic to bacteria than rGO.	[35]
*B. cereus* (G^+^)	HGO	Diffusion plate method (solid nutrient agar)	Composite film containing 15 wt% HGO able to develop clear inhibition zone compared to blank polystyrene film.	[32]

GO (graphene oxide), HGO (hydrophobically modified graphene oxide), FLG (few-layer graphene), Gt (graphite), GtO (graphite oxide), G^+^ (Gram positive), G^−^ (Gram negative).

**Table 2:** Summary for GBNs ability to inhibit fungi.

Microbial Species	GBNs	Methods	Antimicrobial Performance	Ref.
*P. viticola*	GO	Analyze protective effect, fungicidal effect, and curative effect of GO	GO represses germination of sporangia and inhibits development of *P. viticola*.	[36]
*B. sorokiniana*	GO	Observe mycelial and spore growth	GO inhibits spore germination and mycelial growth of *B. sorokiniana* concentration-dependently, and attenuates pathogenicity of pathogenic fungi in vivo.	[37]
*C. albicans*	GO	Determine MIC value using broth microdilution assay	MIC value and MFC of GO is 6.25 and 12.5 μg/mL, respectively.	[38]
	HGO	Diffusion plate method (solid potato dextrose agar)	Composite film containing 15 wt% HGO able to develop clear inhibition zone compared to blank polystyrene film.	[32]
*F. poae*	GO, rGO	Observe mycelial growth, mycelial biomass, and spore germination rate	GO and rGO produce no effect on mycelial growth rate, but decrease hyphae density of *F. poae*.	[39]
*F. graminearum*	GO	Calculate spore germination rate	GO inhibits spore germination and germ-tube elongation of *F. graminearum* dose-dependently.	[34]
	GO, rGO	Observe mycelial growth, mycelial biomass, and spore germination rate	GO and rGO produce no effect on mycelial growth rate, but decrease hyphae density of *F. graminearum*.	[39]
*F. oxysporum*	GO	Calculate spore germination rate	GO inhibits spore germination and germ-tube elongation of *F. oxysporum* dose-dependently.	[34]
	rGO	Observe mycelial growth	rGO inhibits mycelial growth of *F. oxysporum*.	[40]
*A. niger*	rGO	Observe mycelial growth	rGO inhibits mycelial growth of *A. niger*.	[40]
*A. oryzae*	rGO	Observe mycelial growth	rGO inhibits mycelial growth of *A. oryzae*.	[40]
*B. cinerea*	rGO	Measure mycelia diameter in in vitro conditions and measure colony area in whole cut flowers	rGO inhibits mycelial growth of *B. cinerea* significantly in concentrations of 100 and 200 mg/L, but not in 5 and 50 mg/L.	[41]

GO (graphene oxide), rGO (reduced graphene oxide), HGO (hydrophobically modified graphene oxide graphene oxide), MIC (minimum inhibitory concentration), MFC (minimum fungicidal concentration).

**Table 3:** Summary of GBNs effect on soil microbial communities.

Soil Type	Parameter	GBNs	Concentration (mg/g)	Exposure Duration	Effects	Ref.
Urban soil	Total organic carbon	GO	0.1, 0.5, 1	59 days	GO treatment did not significantly change the soil microbial biomass throughout the incubation period.	[65]
Urban soil	Total organic carbon, bacterial community	GO	1	7, 14, 21 days	GN treatment did not significantly change the soil microbial biomass, richness, and diversity of soil microbial communities.	[66]
Farmland soil (benzo [a] pyrene-contaminated soil)	Bacterial community	GO	0.1	90 days	GO had no significant effects on the richness and diversity of bacterial communities in benzo [a] pyrene -contaminated soil.	[67]
Urban soil	Bacterial community	GN	10^−4^, 0.1, 1	20, 39 days	The soybean rhizosphere bacterial community can be significantly phylogenetically and functionally altered in response to GN, especially at the reproductive stage.	[68]
Farmland soil	Bacterial community	GN	0.01, 0.1, 1	4, 21, 60 days	The biomass of the bacterial populations increased significantly after 4 days of GN treatment, but completely recovered after 21 days of treatment.	[69]
Farmland soil	Bacterial and fungal communities	GO	10^−12^, 10^−6^, 10^−3^	7, 14, 30 days	The composition, but not the alpha diversity, of bacterial and fungal communities was significantly influenced by GO at all doses with the exception of the lowest dose on day 14.	[70]
Farmland soil	Bacterial community	GO, rGO, aGO	10^−12^, 10^−6^, 10^−3^	7, 14, 30 days	The bacterial community composition, but not alpha diversity, was altered by all treatments except the 10^−12^ mg/g GO, 10^−12^ mg/g rGO, and 10^−3^ mg/g aGO treatments on day 14 only.	[71]
Farmland soil	Bacterial community	GN, GO	0.1	10, 90 days	Both GN and GO treatments increased the abundance and diversity of soil microbial communities.	[72]
Farmland soil	Bacterial community	GO, rGO	0.05	90 days	The rGO, but not GO, induced a lower bacterial richness than the control. However, GO induced larger changes in the community composition and functions than RGO.	[73]
Farmland soil	Bacterial community	GO	0.05	26 days	GO application significantly decreased the InvSimpson index of the soil bacterial community and the ACE and Chao1 richness estimators of the endophytic bacterial community.	[74]
Farmland soil	Bacterial community	GN	0.3	30, 360 days	The alpha diversity of soil bacterial communities was significantly increased with 30 days of GN exposure, and then significantly decreased after 360 days treatment. Compared to 30 days exposure, 360 days exposure more strongly altered the beta diversity of soil bacterial communities.	[75]
Farmland soil	Bacterial community	PGO	5	90 days	PGO treatment increased the richness and diversity and altered the structure of soil bacterial communities.	[76]
Grassland soil	Bacterial and fungal communities	GN	1	1 year	GN exposure reduced soil DNA and altered bacterial communities, but did not affect soil fungal community profiles.	[77]
Forest soil	Bacterial community	GN	0.01, 0.1, 1	7, 15, 30, 60, 90 days	GN significantly increased the community richness and diversity index as well as the abundances of the bacterial community in a concentration and incubation time-dependent manner.	[78]
Mountain soil (Cd-contaminated soil)	Bacterial community	GO	1, 2	60 days	GO increased the population of some bacteria at the genus level but decreased the diversity of bacterial communities in Cd-contaminated soil.	[79]
Forest farm soil (Cd-polluted Haplic Cambisols)	Bacterial community	GN	0.01, 0.1, 1	15, 30, 45, 60 days	GN increased the richness of bacterial communities in Cd-contaminated soil.	[80]

GN (graphene); GO (graphene oxide); PGO (pristine graphene oxide); rGO (reduced graphene oxide); aGO (ammonia-functionalized graphene oxide); InvSimpson index (the inverse of the classical Simpson’s Diversity Index); ACE (abundance-based coverage estimator); Chao1 richness (Chao1 richness is an estimator, which estimates true number of species in sample).
